# Supplementary material for: The molecular determinants of PABPC-mediated deadenylation rate
Source: bioRxiv. 2026 Jun 8:2026.06.05.728831. Preprint. [Version 1] doi: 10.64898/2026.06.05.728831 (PMC13278162; doi:10.64898/2026.06.05.728831)
Supplement: Supplementary file 1 [file NIHPP2026.06.05.728831v1-supplement-1.pdf]

bioRxiv preprint doi: https://doi.org/10.64898/2026.06.05.728831; this version posted June 8, 2026. The copyright holder for this preprint (which was not certified by peer review) is the author/funder, who has granted bioRxiv a license to display the preprint in perpetuity. It is made available under a CC-BY 4.0 International license.

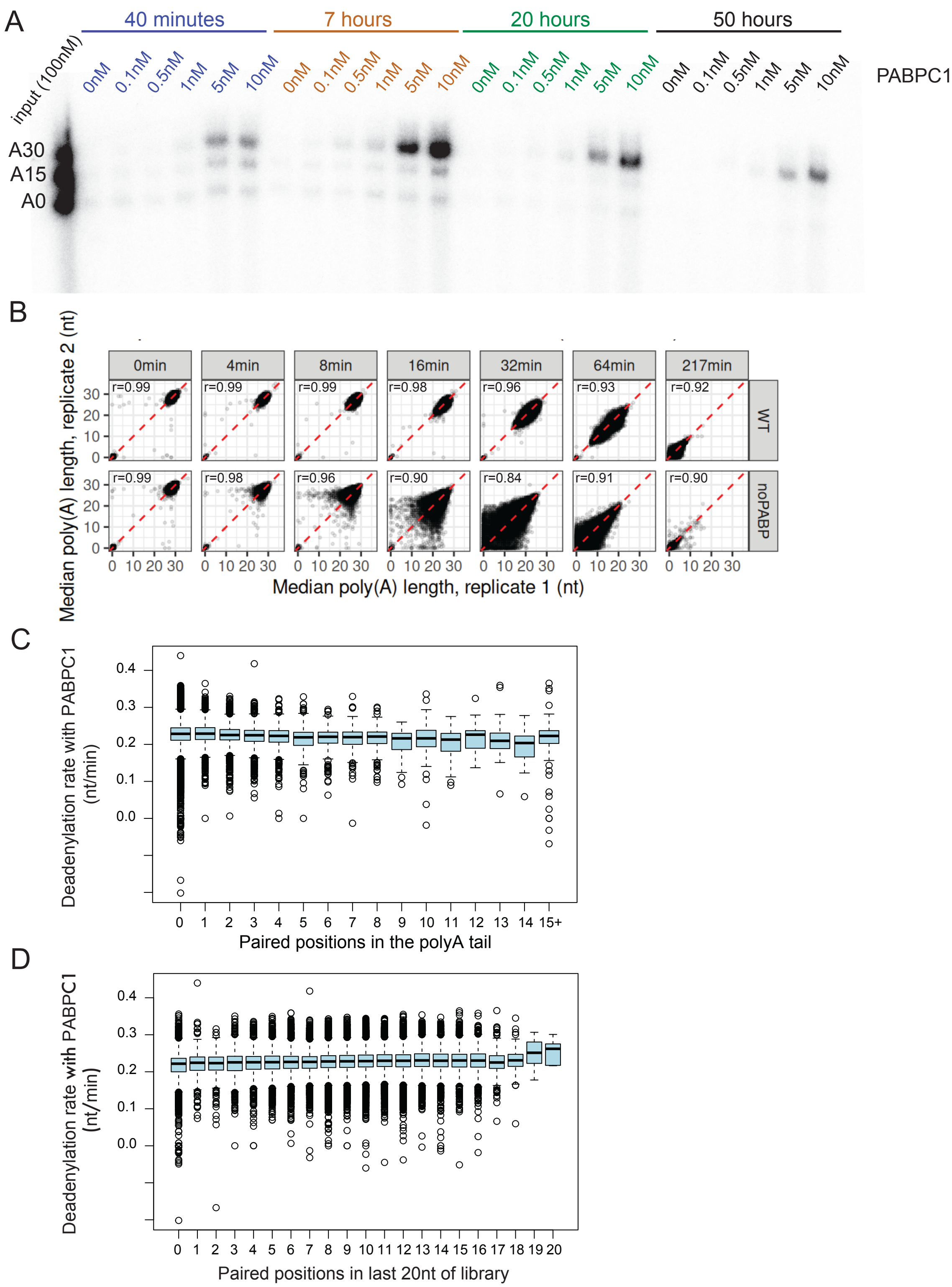

Figure S1. In the presence of PABPC1, UTR structure does not appear to affect deadenylation in vitro. Related to Figure 1.

bioRxiv preprint doi: https://doi.org/10.64898/2026.06.05.728831; this version posted June 8, 2026. The copyright holder for this preprint (which was not certified by peer review) is the author/funder, who has granted bioRxiv a license to display the preprint in perpetuity. It is made available under a CC-BY 4.0 International license.

(A) Time required for PABPC1 to approach binding equilibrium. 100 nM even-ratio mix of A0, A15, and A30 radiolabeled RNA substrate was incubated with titrated levels of PABPC1 for various lengths of time. Filter binding was used to assess RNA binding to PABPC1.

(B) Replicate plots of median poly(A) tail length across deadenylation time points in the presence (top) or absence (bottom) of PABPC1.

(C) In the presence of PABPC1, negligible change in deadenylation rates as tail residues predicted to be paired increase. Box and whisker plots show the distributions of fitted deadenylation rates in the presence of PABPC1 for library molecules with the indicated number of poly(A)-tail positions that are predicted to be paired in the lowest-energy structure (line, median; box quartile; whisker, 1.5-times IQR rule via Tukey's method).

(D) In the presence of PABPC1, negligible change in deadenylation rates of molecules with more tail-proximal residues that are predicted to be paired. This panel is as in A, but instead groups deadenylation rates by the number of positions within 20 nt preceding the poly(A) tail that are predicted to be paired in the lowest-energy structure.

bioRxiv preprint doi: https://doi.org/10.64898/2026.06.05.728831; this version posted June 8, 2026. The copyright holder for this preprint (which was not certified by peer review) is the author/funder, who has granted bioRxiv a license to display the preprint in perpetuity. It is made available under a CC-BY 4.0 International license.

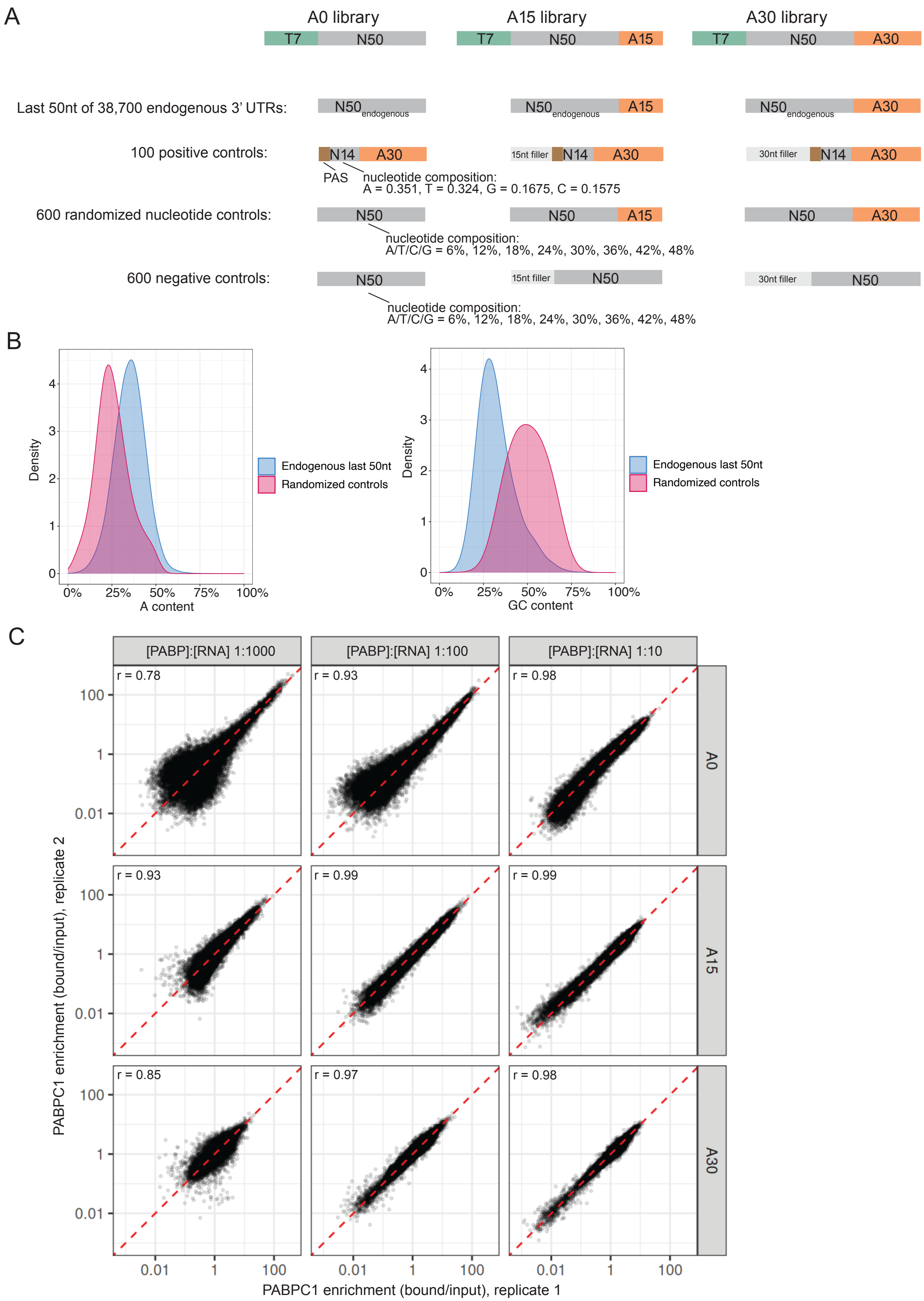

bioRxiv preprint doi: https://doi.org/10.64898/2026.06.05.728831; this version posted June 8, 2026. The copyright holder for this preprint (which was not certified by peer review) is the author/funder, who has granted bioRxiv a license to display the preprint in perpetuity. It is made available under a CC-BY 4.0 International license.

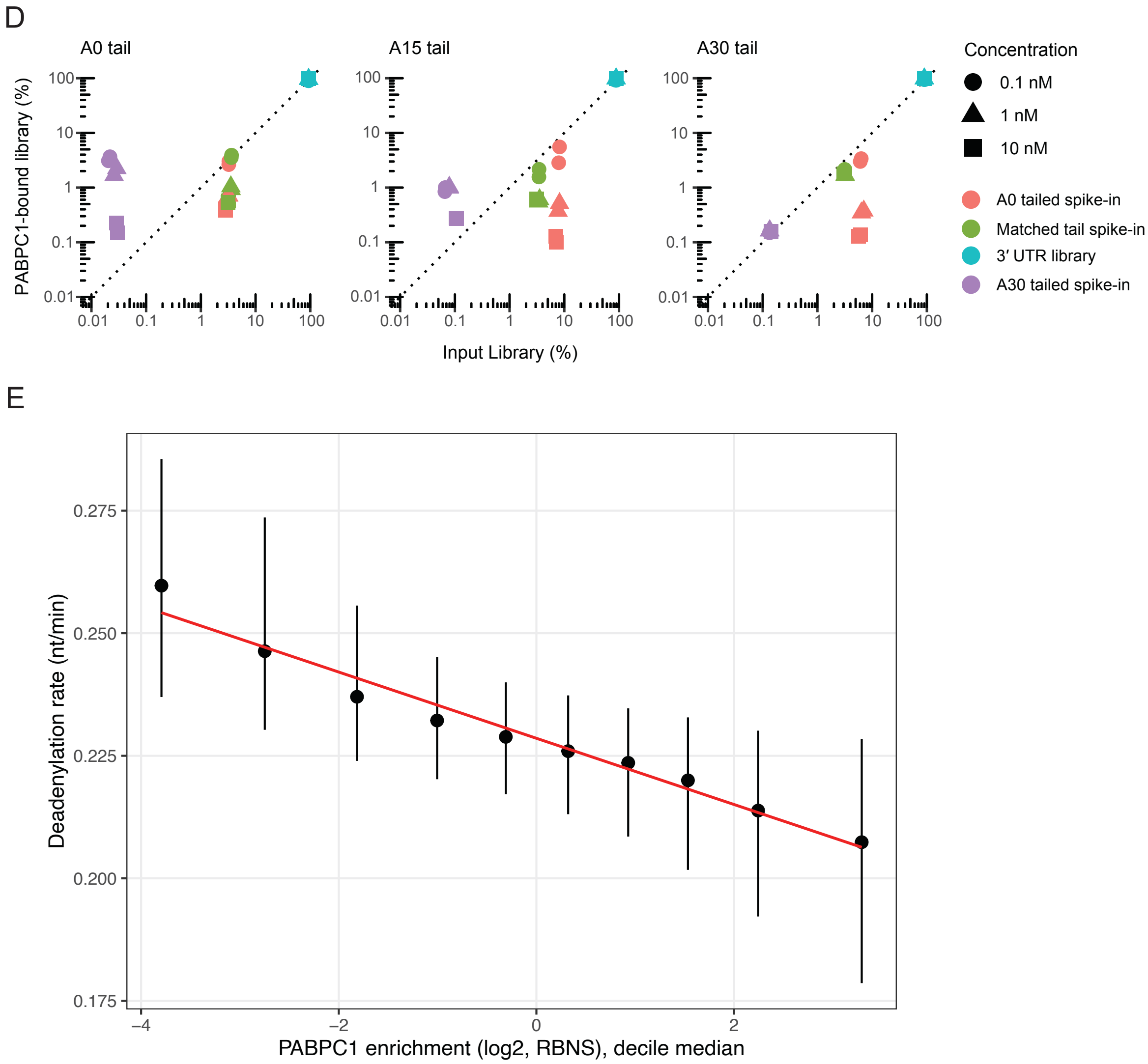

Figure S2. RNA substrate spike-in design and bulk PABPC1 binding propensity properties. Related to Figure 2.

(A) RNA library design with spike-in controls. Positive controls contained an A_{30} tail, and negative controls lacked a poly(A) tail. $N50_{endogenous}$ represent human UTR sequences, whereas N14 and N50 regions are random-sequence regions that were then filtered to a set that achieved the specified nucleotide composition. N14 sequences had the same nucleotide composition as human 3′ UTRs, whereas N50 sequences had a spread of nucleotide compositions such that at least 20 sequences represented each nucleotide at each of the specified percentages (6, 12, 18, 24, 30, 36, 42, 48).

(B) The distribution of A-content and GC-content across the designed library and randomized control sequences.

bioRxiv preprint doi: https://doi.org/10.64898/2026.06.05.728831; this version posted June 8, 2026. The copyright holder for this preprint (which was not certified by peer review) is the author/funder, who has granted bioRxiv a license to display the preprint in perpetuity. It is made available under a CC-BY 4.0 International license.

(C) Replicate plots of PABPC1 enrichment (bound/input) from RBNS across tail lengths and PABPC1 concentrations.

(D) Enrichment plots comparing the input library against the PABPC1-bound library. PABPC1 concentration titration is depicted as circle, triangle, or square (key) and sample descriptors are depicted with orange, green, blue, or purple color.

(E) Magnitude of effect visualized by quantile-binned comparison of deadenylation rate in the presence of PABPC1 as a function of PABPC1 RBNS enrichment.

bioRxiv preprint doi: https://doi.org/10.64898/2026.06.05.728831; this version posted June 8, 2026. The copyright holder for this preprint (which was not certified by peer review) is the author/funder, who has granted bioRxiv a license to display the preprint in perpetuity. It is made available under a CC-BY 4.0 International license.

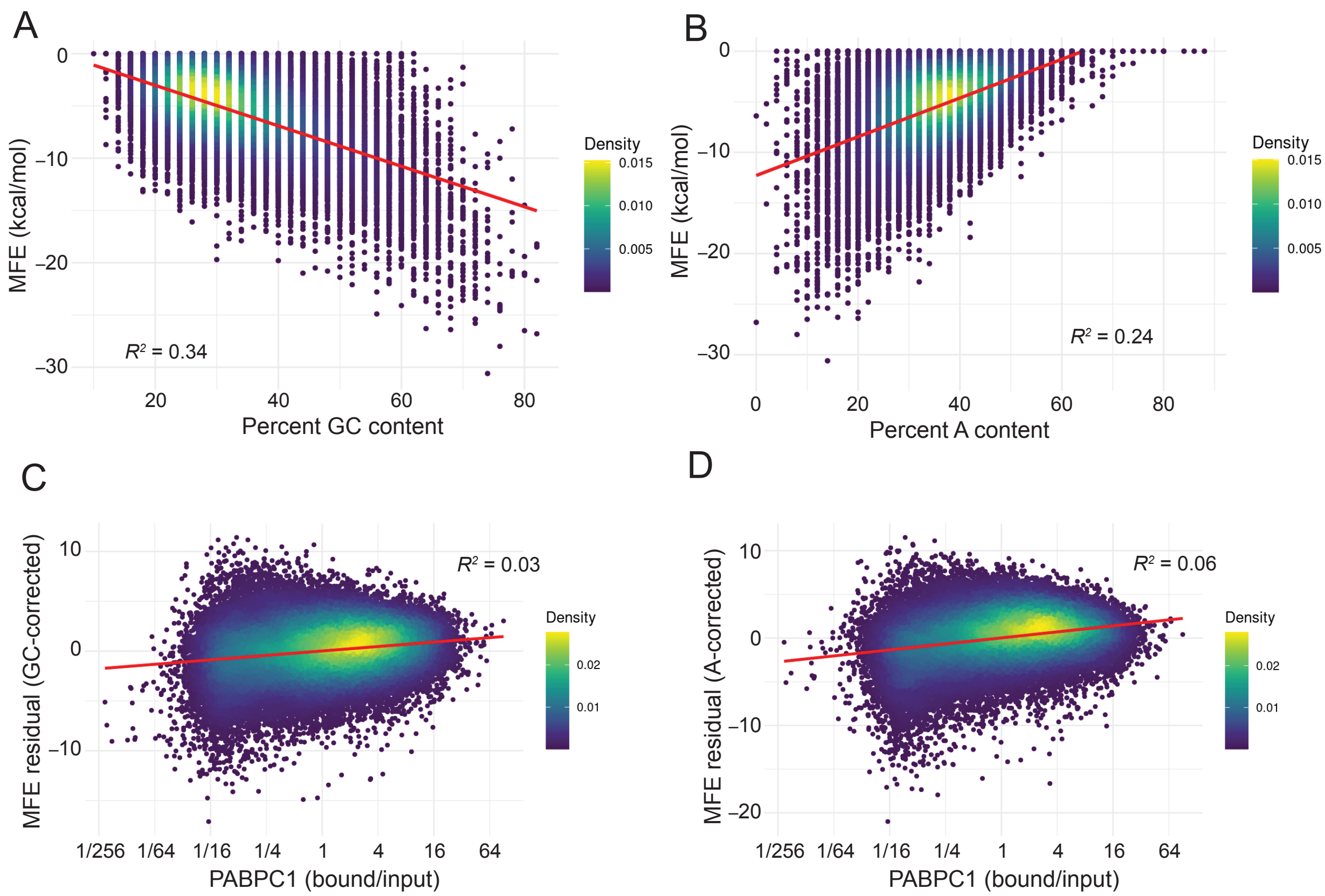

Figure S3. MFE residual after correction for nucleotide content shows little correlation with PABPC1 apparent affinity. Related to Figure 3.

(A) Negative correlation between MFE (calculated for each 50 nt UTR with adjoining A_{15} tail) and percent GC content. Each point in the scatterplot represents a member of the RNA library.

(B) Positive correlation between MFE and percent A content; otherwise in A.

(C) Negligible correlation between MFE with GC-content correction and PABPC1 apparent affinity, measured as enrichment in the PABPC1-bound fraction (A_{15} library at 1 nM PABPC1).

(D) Poor correlation between MFE with A-content correction and PABPC1 apparent affinity; otherwise, as C.

bioRxiv preprint doi: https://doi.org/10.64898/2026.06.05.728831; this version posted June 8, 2026. The copyright holder for this preprint (which was not certified by peer review) is the author/funder, who has granted bioRxiv a license to display the preprint in perpetuity. It is made available under a CC-BY 4.0 International license.

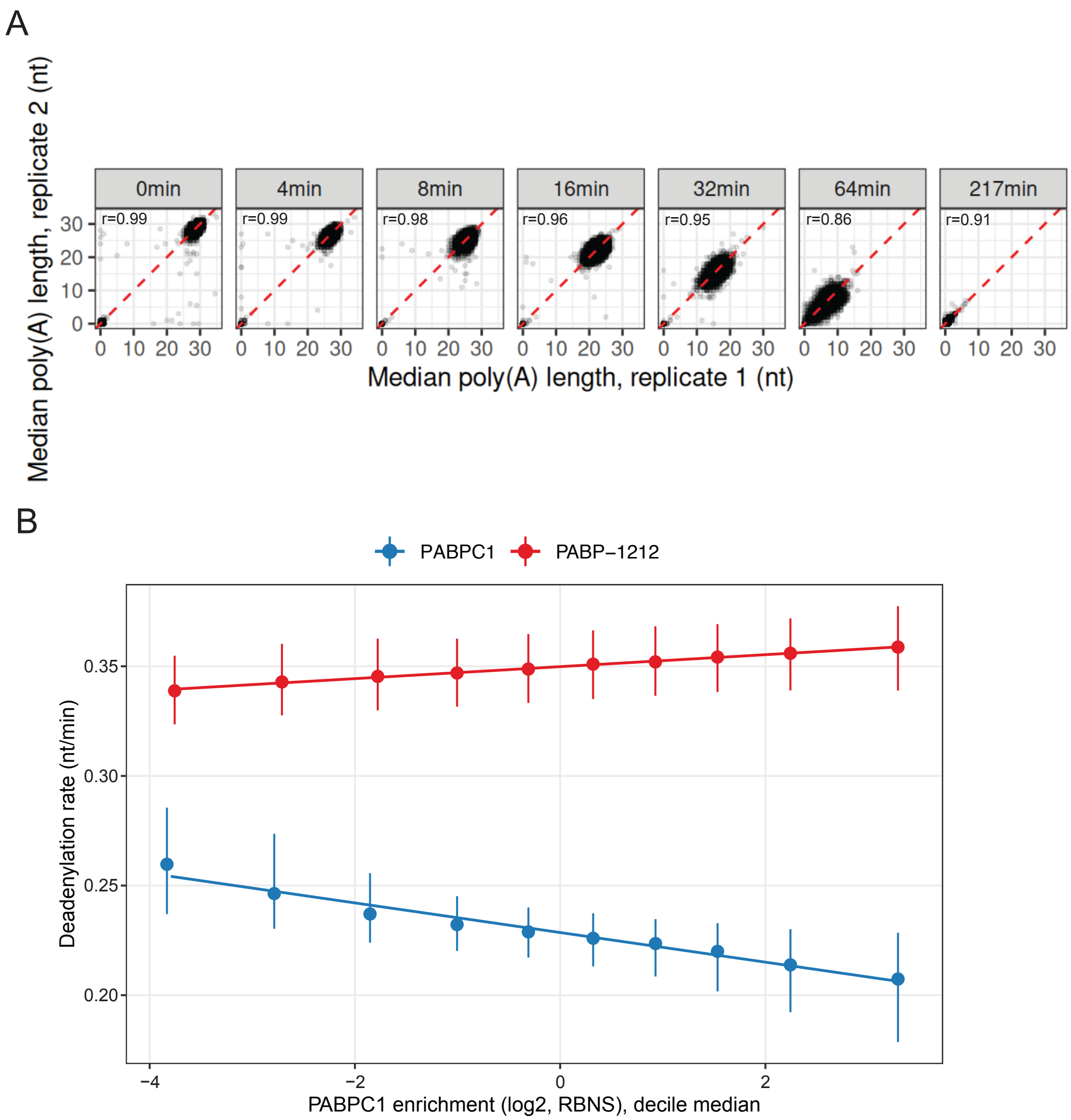

Figure S4. A PABPC1 domain-swap mutant that loses UTR binding also loses sequence-associated deadenylation protection. Related to Figure 4.

(A) Replicate plots of median poly(A) tail length across deadenylation time points in the presence of PABP-1212.

(B) Magnitude of effect visualized by quantile-binned comparison of deadenylation rate in the presence of PABP-1212, compared to PABPC1, as a function of PABPC1 RBNS enrichment.
